# A moonlighting metabolic protein influences repair at DNA double-stranded breaks

**DOI:** 10.1093/nar/gku1405

**Published:** 2015-01-27

**Authors:** Ana Lilia Torres-Machorro, John P. Aris, Lorraine Pillus

**Affiliations:** 1 Section of Molecular Biology, Division of Biological Sciences, UC San Diego Moores Cancer Center, University of California, San Diego, La Jolla, CA 92093-0347, USA; 2 Department of Anatomy and Cell Biology, Health Science Center, University of Florida, Gainesville, FL 32610-0235, USA

## Abstract

Catalytically active proteins with divergent dual functions are often described as ‘moonlighting’. In this work we characterize a new, chromatin-based function of Lys20, a moonlighting protein that is well known for its role in metabolism. Lys20 was initially described as homocitrate synthase (HCS), the first enzyme in the lysine biosynthetic pathway in yeast. Its nuclear localization led to the discovery of a key role for Lys20 in DNA damage repair through its interaction with the MYST family histone acetyltransferase Esa1. Overexpression of Lys20 promotes suppression of DNA damage sensitivity of *esa1* mutants. In this work, by taking advantage of *LYS20* mutants that are active in repair but not in lysine biosynthesis, the mechanism of suppression of *esa1* was characterized. First we analyzed the chromatin landscape of *esa1* cells, finding impaired histone acetylation and eviction. Lys20 was recruited to sites of DNA damage, and its overexpression promoted enhanced recruitment of the INO80 remodeling complex to restore normal histone eviction at the damage sites. This study improves understanding of the evolutionary, structural and biological relevance of independent activities in a moonlighting protein and links metabolism to DNA damage repair.

## INTRODUCTION

The characterization of a gene or protein generally focuses on the context in which it first appeared; thus, distinct functions of proteins are often unsuspected. Dual or moonlighting functions for a single protein have central implications in the evolution of complex processes and can provide insight into regulatory mechanisms that connect cellular pathways and functions (1). Recognition of moonlighting proteins is increasing (reviewed in (1–5)). Consider the following examples: the τ-crystallin is a major structural protein of the eye lens, yet it also acts as an enolase to catalyze a step in glycolysis (6). The mammalian NCOAT enzyme is an O-GlcNase, which removes a carbohydrate modification from proteins, yet it is also a histone acetyltransferase (HAT) affecting chromatin regulation (7).

In yeast, Lys20 and its isozyme Lys21 are defined as homocitrate synthases (HCS). These enzymes catalyze the first committed and rate-limiting step of the α-aminoadipate lysine biosynthesis pathway of fungi (8). Early biochemical data identified HCS as a mitochondrial protein (9,10). However, cell biological and refined biochemical studies revealed that Lys20 has a predominant nuclear localization (11,12). Despite these reports, a distinct nuclear role of Lys20 remained speculative for many years.

A clue to the nuclear function of Lys20 came from studies on the essential HAT Esa1, a homolog of human Tip60 (13,14). Esa1 preferentially acetylates histones H2A and H4 (13,14), the histone variant Htz1 (15–17) and more than 200 non-histone substrates (18,19). It participates in DNA damage repair through at least two independent mechanisms: transcriptional regulation of DNA damage-induced gene expression (20,21) and localized signaling at DNA double-stranded breaks (DSBs) (22), where it transiently acetylates histone H4 as part of the signal transduction pathway leading to ligation of broken DNA ends (23). Thus, cells with conditional alleles of *esa1* are sensitive to DNA damage (22,24).

In a genetic screen, increased *LYS20* gene dosage was found to suppress the DNA damage sensitivity of *esa1* mutants (25,26). A *lys20-E155A* mutant is unable to catalyze lysine synthesis, yet it still suppresses *esa1* DNA damage sensitivity, suggesting that Lys20 has a second, moonlighting function in DNA damage repair (26). Even though the HCS activity of Lys20 is unnecessary to suppress *esa1*, nuclear localization of Lys20 is essential for suppression (26).

Mutants affecting the function of the chromatin remodeling complex INO80 are also sensitive to DNA damage (27,28). INO80 has multiple roles in chromatin-regulated processes, including gene expression (27,29), chromosome stability (30) and DNA damage repair (31). INO80 is recruited directly to DSBs to promote histone eviction early in repair (32). Histone eviction is necessary for activation of the Mec1-dependent checkpoint (33) and later recruitment of the key repair factors Rad51 and Rad52 (32). During repair, INO80 is also important for increasing chromatin mobility (34) and for strand invasion by promoting histone eviction at the donor sequence (35).

In this work we report characterization of molecular elements of suppression of *esa1* by Lys20 overexpression. The moonlighting domain that promotes DNA repair was pinpointed to the C-terminal region of Lys20. The metabolically inactive moonlighting protein was found to be recruited to sites of DNA damage, having increased levels of recruitment when overexpressed. Following break induction, *esa1* mutants had impaired histone acetylation at sites of DNA damage that was accompanied by compromised histone eviction, consistent with DNA damage sensitivity. Lys20 overexpression suppressed *esa1* by promoting increased accumulation of the INO80 remodeling complex at the breaks to mediate normal histone eviction in *esa1* mutants.

## MATERIALS AND METHODS

### Yeast strains and plasmids

Yeast strains, plasmids and oligonucleotides are listed in Supporting Information: Supplementary Tables S1, S2 and S3. The *esa1–414* allele was previously described (14). The *INO80–9MYC-TRP1* cassette was amplified from strain MAO104 (kindly donated by M.A. Osley) (32) to replace endogenous *INO80* in the W303 background. The strains generated were LPY20339, 20531, 20539 and 20541. All other mutants are null alleles constructed with standard methods. Site-directed mutagenesis was performed in the plasmid containing wild-type *LYS20* (pLP1412) with standard methods and primers listed in Supplementary Table S3. Accurate mutagenesis was verified by sequencing.

### Growth assays

Dilution assays were performed as described (24). They represent 5-fold serial dilutions from 0.5 A_600_ units of cells. Cultures were grown to saturation before plating. Camptothecin (CPT) sensitivity was assayed using 7, 20 or 30 μg/ml in DMSO, added to YPAD or ura- drop out plates buffered with 100-mM potassium phosphate to pH 7.5 (36). The specific concentration used is noted in each figure and was selected to capture the greatest dynamic range for the conditions being tested. Images were taken after 2–6 days.

### Immunoblots

Whole cell extracts were prepared by bead beating as described (14). Subcellular fractionation was done through a series of differential centrifugation steps (37). To crosslink, samples were treated 15 min with 1% formaldehyde. Proteins were separated on sodium dodecyl sulphate-polyacrylamide gel electrophoresis (SDS-PAGE), transferred to nitrocellulose and probed with: anti-Lys20 (1:5000, mAb 40C4, that is equivalent to the published mAb C65) (11), anti-Myc (1:5000, 9E10.2) (38), anti-H4K8Ac (1:2000, Millipore), anti-H4K12Ac (1:2000, Active Motif), anti-H3Ct (1:10,000, Millipore), anti-H3K9, K14Ac (1:10,000, Upstate), anti-Sir2 (1:10,000) (39), anti-Pgk1 (1:10,000) and anti-β Tubulin (1:20000) (40).

### HO induction and chromatin immunoprecipitation

Cells were grown in YP or ura- media containing 2% raffinose to A_600_ = 0.5 at 30°C. Fifty milliliters were removed at time zero and processed for chromatin immunoprecipitation (ChIP); galactose was then added to the culture (2% final concentration) to induce expression of HO (41). Cells were grown for two or three more hours and samples were retrieved 1, 2 and 3 h after galactose induction for ChIP sample preparation. For repair induction, cells were collected after 2 h in galactose (23). After centrifuging, the pellet was resuspended in pre-warmed ura- media containing 2% glucose to promote repression of HO.

ChIP was performed as described previously (42). Crosslinking was with 1% formaldehyde for 15 min at room temperature (RT). Sheared chromatin was incubated overnight at 4°C with 1:400 anti-Lys20 (40C4), 1:200 anti-Myc (9E10.2), 1:500 anti-H4K5Ac (Millipore), 1:500 anti-H4K16Ac (Millipore) or 1:500 anti-H3-CT (Millipore). Protein G-sepharose beads were used for anti-Myc ChIPs and protein A-sepharose beads were used for the other antibodies. After treating with RNase and proteinase K, and reversing the crosslinking at 65°C, the DNA was purified with the QIAquick PCR purification kit (Qiagen). Input DNA and immunoprecipitation (IP) samples were diluted 50 and 10-fold, respectively, and were analyzed by real-time polymerase chain reaction (PCR) using primers and probes (listed in Supplementary Table S3) on a DNA Engine Opticon 2 (MJ Research). ChIP samples of cells derived from strain JKM179 (Figure 3C and D) (43) were analyzed with SYBR green with primers localized 0.2 kb downstream of the break (Supplementary Table S3). Repairing strains (Figure 3E and F, Figure 4, and Figure 5B and C), encoding the silent mating-type loci derived from strain BAT009 (23) were analyzed with published primers and probes that recognize a region 0.6 kb downstream of the HO site (Supplementary Table S3). Quantitative PCRs (qPCRs) performed with specific probes prevented noise derived from the silent mating-type loci (23). The probes in this study were synthesized by Eurofins MWG Operon. The real-time PCR mixes (for SYBRgreen and probes) were purchased from Anaspec. Results shown are the average of triplicates from three independent experiments.

**Figure 1.**
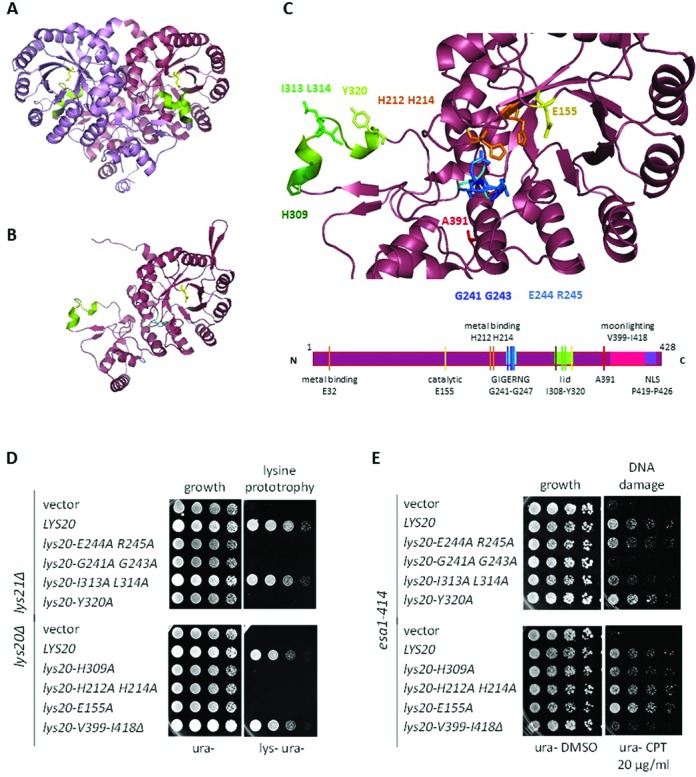
The Lys20 moonlighting function localized to a 20 amino acid C-terminal domain. (**A**) Ribbon diagram of an HCS homodimer depicting Lys4, the *Schizosaccharomyces pombe* homolog of Lys20 (44). The monomers are distinguished by coloring: violet and maroon. (**B**) Ribbon diagram of a Lys20 monomer. The *S. cerevisiae* Lys20 structure was modeled with the *PyMOL* Molecular Graphics System, using the crystal structure of Lys4 of *S. pombe* as a template. The lid domain is represented in green. (**C**) Localization of the residues evaluated in the structure-function assays that identified the moonlighting domain of Lys20. The localization of the color-coded residues in a linear cartoon of the protein is shown below. E155 is a previously characterized catalytic amino acid of Lys20. Of the three amino acids necessary for metal binding (E32, H212 and H214), H212 and H214 were tested. GIGERNG is a highly conserved domain that is found close to the catalytic region. Because the crystal structure of the template Lys4 terminated at A391, the moonlighting domain and the NLS of Lys20 could not be modeled. (**D**) Residues I313, L314 and V399 to I418 were not necessary for the biosynthetic activity of Lys20. Growth assays of *lys20Δ lys21Δ* strains transformed with *LYS20* mutants. Growth on medium without lysine indicated that the strain was proficient for HCS catalytic activity. (**E**) Residues V399 to I418 encompassed the moonlighting domain of Lys20. Growth assay of *esa1–414* strains plated on medium with the DSB-inducing drug camptothecin (CPT) or on the vehicle control DMSO. Suppression of the DNA damage sensitivity of *esa1–414* by increased *LYS20* gene dosage was used as a control to analyze the ability of *lys20* mutants in (D) to suppress *esa1–414*. The *lys20-V399-I418Δ* (*lys20-moon*) mutant was unable to suppress *esa1–414* DNA damage sensitivity, yet remained proficient for lysine synthesis.

**Figure 2.**
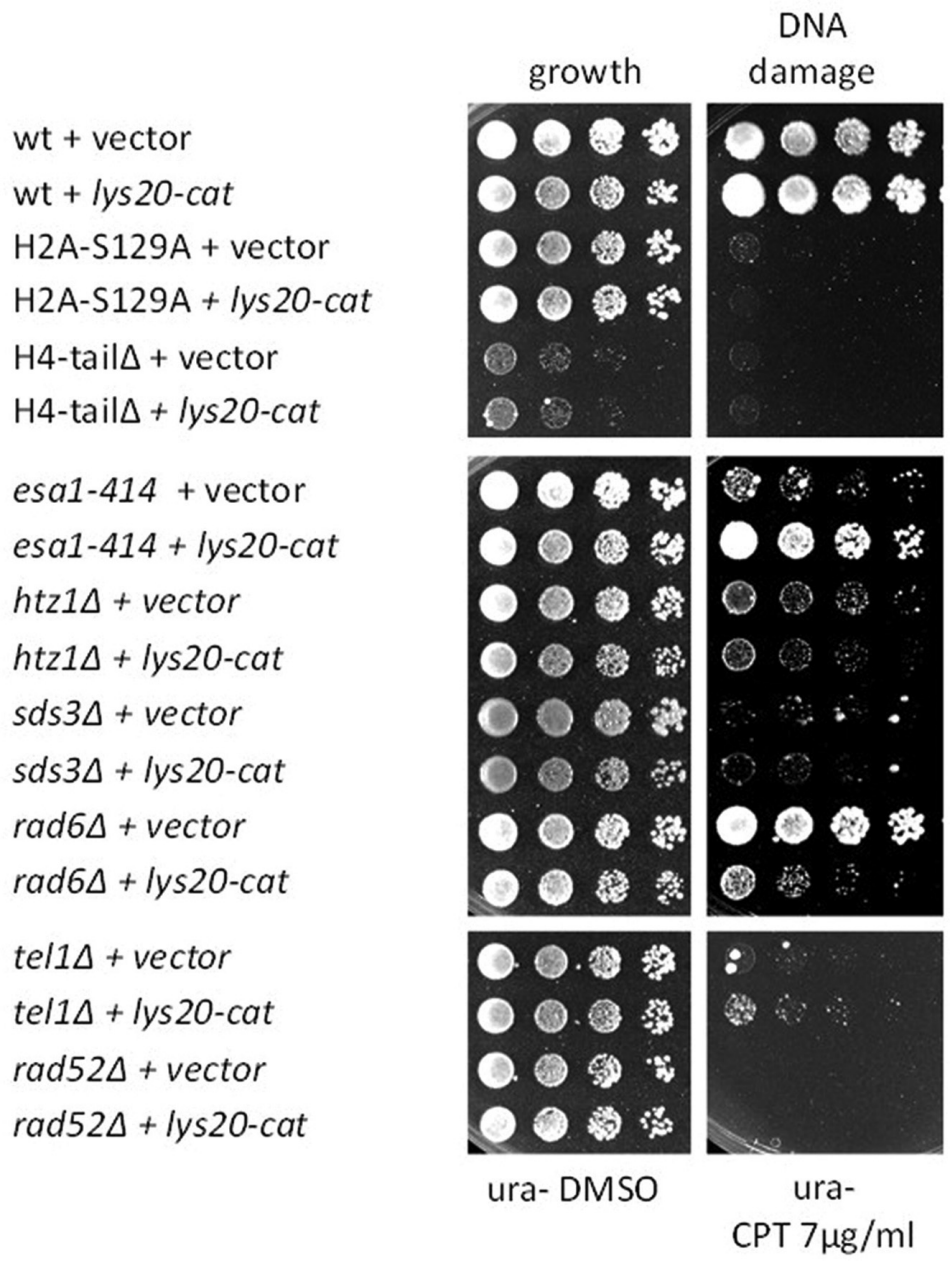
*LYS20* interacted genetically with DNA damage response genes. DNA damage sensitive strains were transformed with vector or with the *lys20-cat* mutant (*lys20-E155A*) that was competent for moonlighting activity but not for lysine biosynthesis. Increased dosage of *lys20-cat* improved the DNA damage sensitivity of *esa1–414* and *tel1Δ*, but interfered in *rad6Δ* cells. The *lys20-cat* mutant was used because it was more proficient in suppressing *esa1–414* compared to wild-type *LYS20* (Figure 1E).

**Figure 3.**
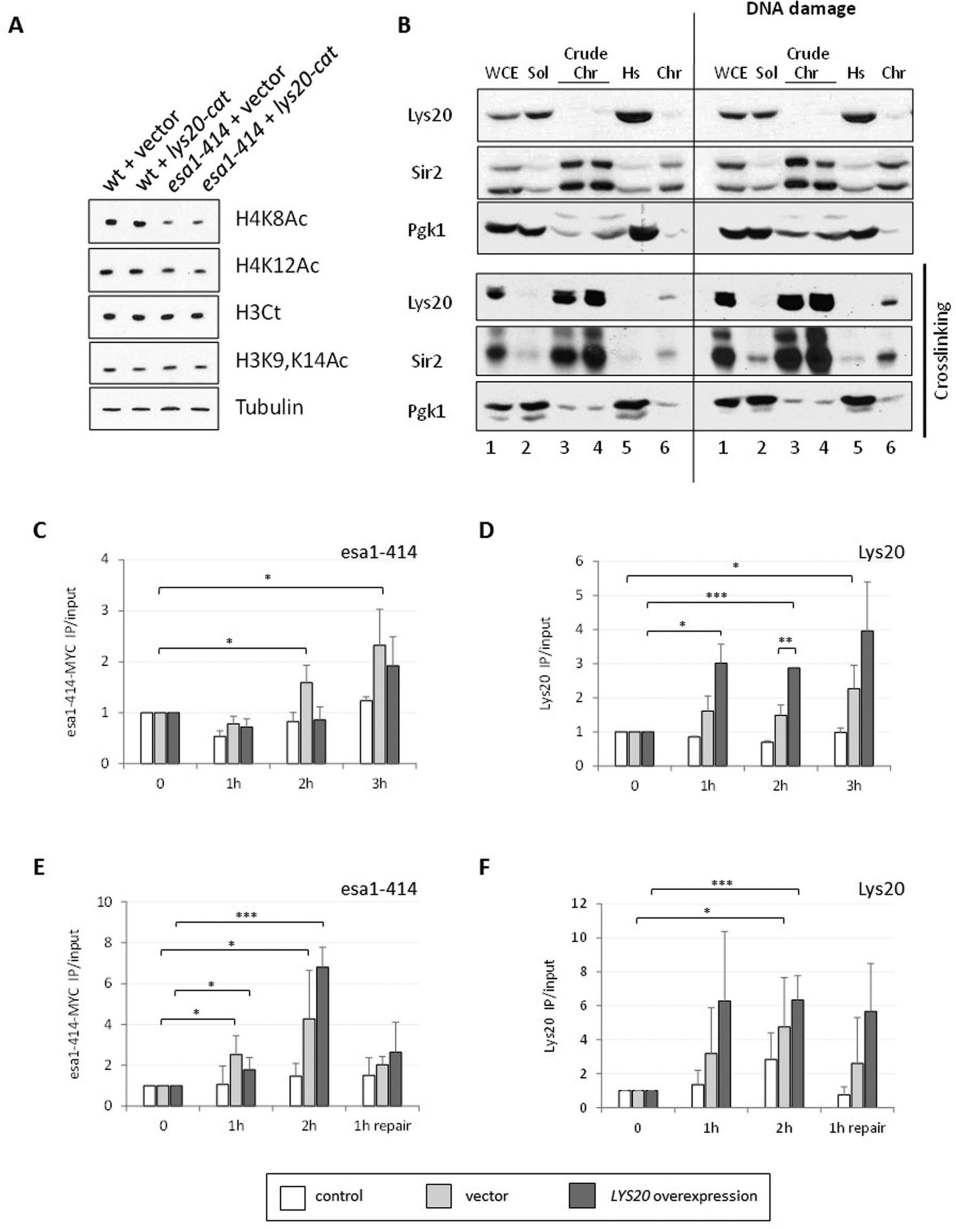
Lys20 was recruited to DNA DSBs with similar kinetics to the HAT Esa1. (**A**) Increased *lys20-cat* dosage did not promote increased global histone H4 acetylation. Protein lysates were probed as indicated. Tubulin was included as a loading control. (**B**) Subcellular fractionation assays revealed that Lys20 binding to chromatin was transient or unstable. The protocol consisted of lysing cells to obtain crude chromatin (Crude Chr) and soluble fractions (Sol). A portion of the crude chromatin fraction was briefly treated with micrococcal nuclease to release polynucleosomes, which were collected with an ultracentrifugation step (Chr). Fractions tested were: whole cell lysate (W), soluble fraction (S), crude chromatin (Crude Chr), high-speed supernatant (Hs) and Chromatin (Chr). The cells were untreated or treated for 90 min with 0.1-M hydroxyurea to induce DNA damage prior to lysate preparation. Anti-Sir2 recognizes two specific bands not observed in a *sir2Δ* strain, the more rapidly migrating of which is likely to be an N-terminal proteolytic product, since the antiserum was raised to the 13 C-terminal amino acids of Sir2 (not shown). (**C**) Myc-tagged *esa1–414* was recruited 0.2 kb downstream of the DSB at 2 and 3 h after break induction. ChIP anti-Myc of a control untagged strain (white) and of an *esa1–414–13MYC* tagged strain transformed with vector (light gray) or overexpressing *LYS20* (dark gray). The enrichment of Myc-tagged esa1–414 relative to input and to the control locus *SCR1* is shown. Time points in all ChIP experiments were time 0 (no HO induction), 1 h of growth in galactose (induction of HO), 2 h in galactose and 3 h in galactose. (**D**) Lys20 overexpression promoted recruitment 0.2 kb downstream of the break. ChIP anti-Lys20 in the same strains as (C), with control (white) representing a no-antibody control. The enrichment of Lys20 is shown relative to input and to *SCR1*. (**E**) Esa1 was enriched 0.6 kb downstream of the DSB 1 and 2 h after break induction. Esa1 no longer bound after repair had started. ChIP anti-Myc in an *esa1–414–13MYC* tagged strain that can repair by HR because the chromosomal silent mating-type loci are present. The control (white) was an untagged *lys20Δ lys21Δ* strain, whereas light gray and dark gray columns corresponded to the Myc-tagged strain transformed with vector or with 2μ *LYS20*, respectively. The enrichment relative to input and to the control locus *SMC2* was graphed. Time points tested were as in (C), except with the last time point as 1 h of growth in glucose medium, to repress HO and allow repair by HR. (**F**) Endogenous Lys20 was recruited to the DSB, however higher levels were recruited when the protein was overexpressed. ChIP anti-Lys20 in the same strains as (E). The control (white) is a *lys20Δ lys21Δ* strain. The vector strain had endogenous levels of Lys20 expression, whereas the *LYS20* sample overexpressed *LYS20*. Data represent triplicate samples for three independent experiments. Error bars represent the standard deviation (SD). The student's *t*-test was used to assess statistical significance and was represented with asterisks as follows: *P < 0.05, **P < 0.01 and ***P < 0.001.

**Figure 4.**
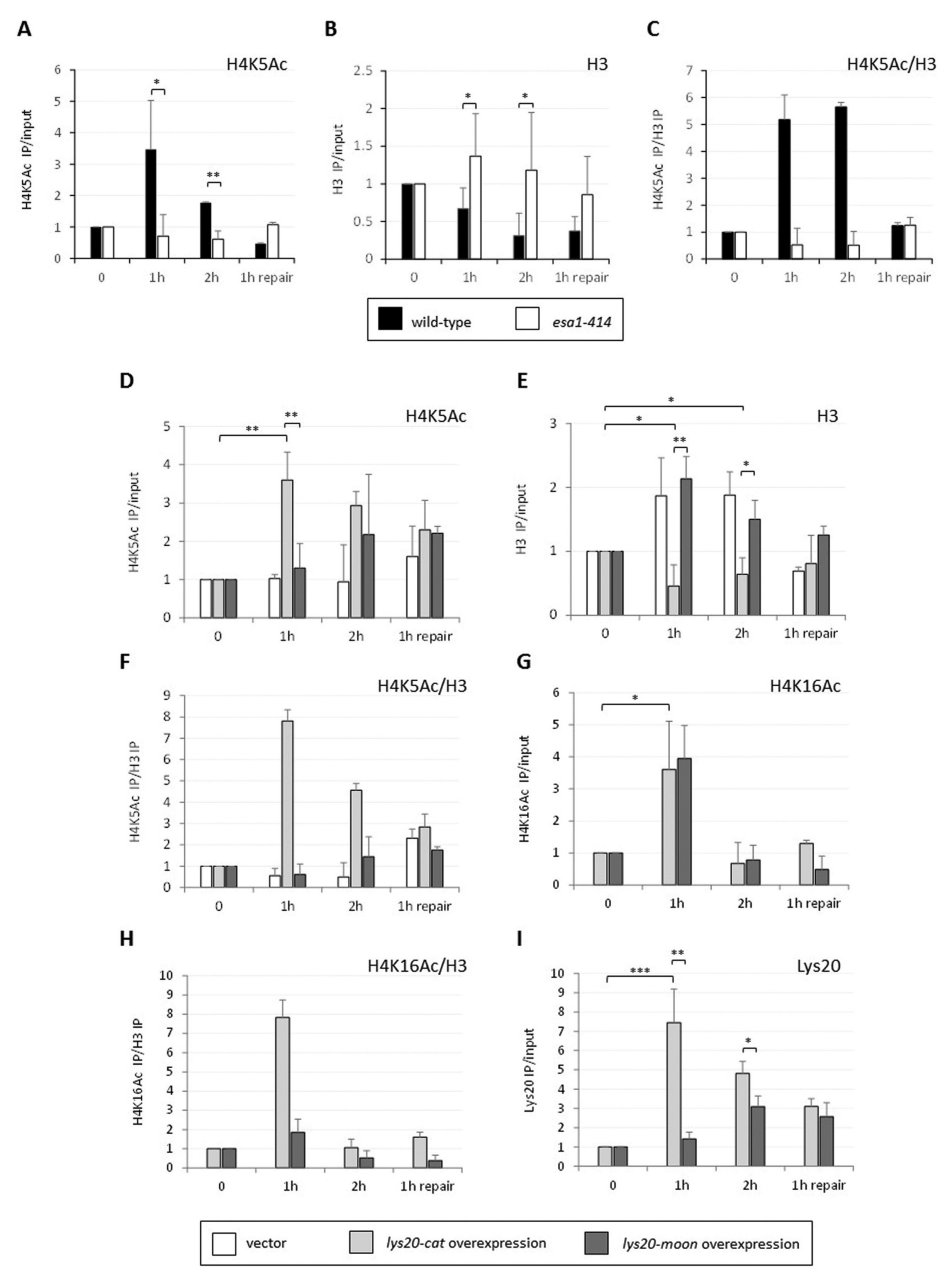
The *esa1–414* cells had defective histone acetylation and eviction at the DSB. (**A**) Histone H4K5 acetylation was defective in *esa1–414* cells compared to wild-type. ChIP anti-H4K5Ac in wild-type and *esa1–414* strains. (**B**) Histone eviction at the break was impaired in *esa1–414* cells. ChIP anti-H3Ct in strains in (A). (**C**) Relative H4 acetylation to histone levels increased after break induction in wild-type but not in *esa1–414* cells. (**D**) Localized H4K5 acetylation increased when *lys20-cat* was overexpressed in *esa1* cells 1 and 2 h after break induction. ChIP anti-H4K5Ac in *esa1–414 lys20Δ lys21Δ* strains transformed with vector (white), *lys20-cat* (light gray) or *lys20-moon* (dark gray). Time points and normalization are as in Figure 3E. (**E**) The histone eviction defect in *esa1–414* cells was rescued by *lys20-cat* overexpression. Histone H3 levels were tested in the same strains as in (D) with anti-H3Ct ChIPs before, during and after HO break induction. (**F**) H4K5 acetylation relative to histone levels dramatically increased when *lys20-cat* was overexpressed. The enrichment of H4K5 acetylation at the break is shown relative to histone levels. Because histone H4 and H3 are heterodimers in DNA (52), comparison of histone H4 acetylation relative to histone H3 reflects histone/nucleosome occupancy. (**G**) Histone H4K16 acetylation increased equally in *esa1* strains transformed with *lys20-cat* and *lys20-moon*. Histone H4K16 acetylation was tested in the same strains as in (D). (**H**) Relative histone H4K16 acetylation only increased when *lys20-cat* was overexpressed. H4K16 acetylation is shown relative to histone levels. (**I**) The moonlighting domain of Lys20 was important for recruitment to the DSBs. ChIP anti-Lys20 in a *lys20Δ lys21Δ* strain expressing *lys20-cat* or *lys20-moon*. Data represent three independent experiments. SD and *P*-values are the same as in Figure 3.

**Figure 5.**
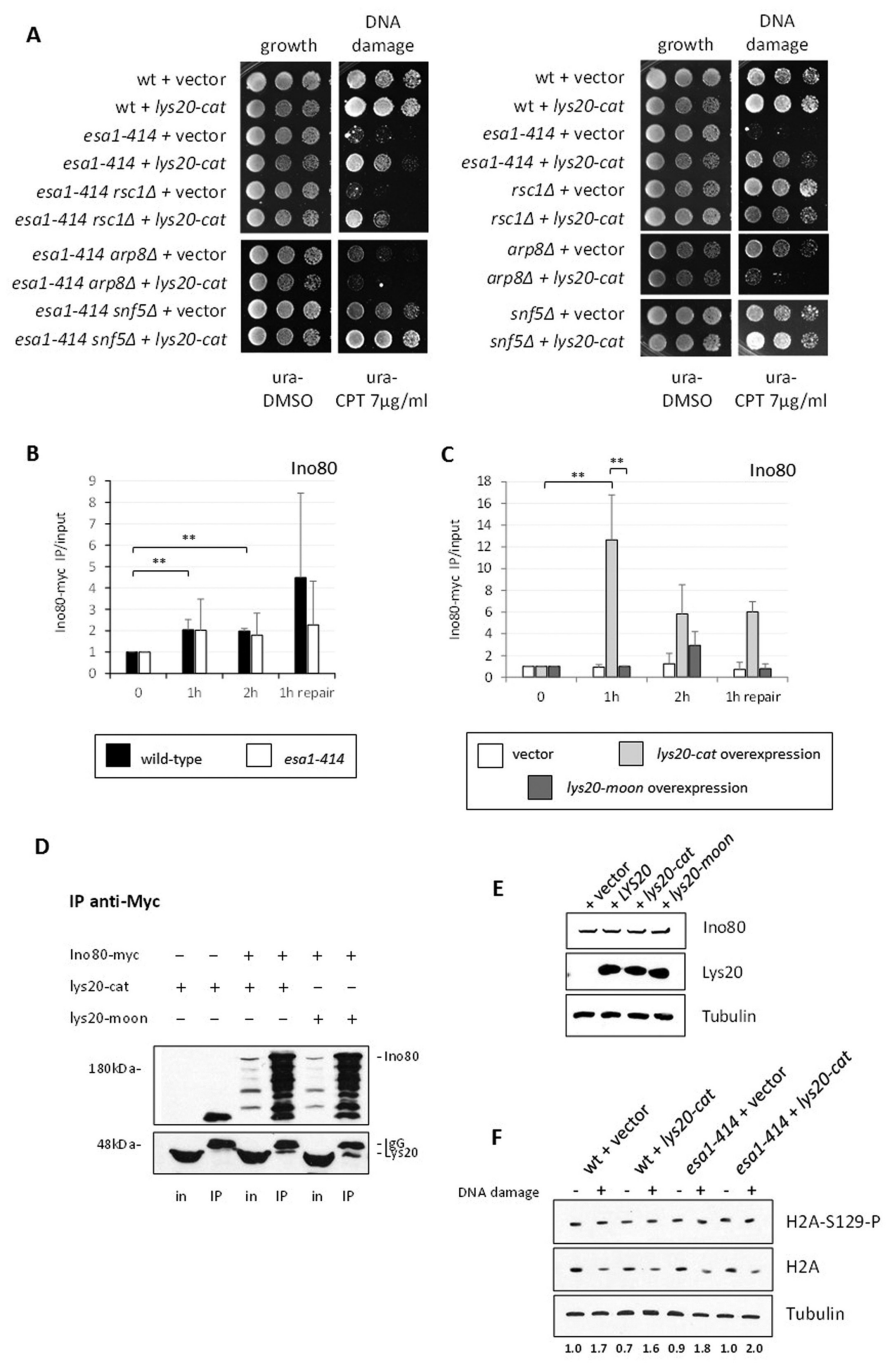
*LYS20* overexpression enhanced INO80 recruitment in *esa1* cells. (**A**) Suppression of *esa1* by Lys20 overexpression was dependent on the INO80 complex. Double mutants combining *esa1–414* with mutants impairing different remodeling complexes were tested for suppression by *LYS20* overexpression. Single mutant controls are indicated on the right. Deletion of catalytic subunits of INO80 and RSC is lethal (32,57). The *ino80Δ* strain is viable in the S288c background, but not in W303, which is used here (27). The INO80 complex (assayed using *arp8Δ*) proved necessary for suppression by Lys20 as increased dosage of Lys20 antagonized repair when the INO80 complex was disrupted, a result also observed in the single *ARP8* null. (**B**) Ino80 recruitment to the break was similar in wild-type and *esa1* cells. ChIP anti-Myc in *INO80–9MYC* and *esa1–414 INO80–9MYC* strains. (**C**) Overexpression of *lys20-cat* promoted increased recruitment of Ino80, 1 and 2 h after break induction in *esa1* cells. ChIP anti-Myc in *esa1–414 INO80–9MYC lys20Δ lys21Δ* strains transformed with vector, *lys20-cat* and *lys20-moon*. Normalization, SD and *P*-values are the same as in Figure 3. (**D**) Ino80-myc and Lys20 interacted physically. When Ino80 is precipitated with an anti-Myc antibody, lys20-cat and lys20-moon co-immunoprecipitate. Input (in) and immunoprecipitated (IP) fractions are shown. The first two lanes in each blot were prepared from a strain expressing untagged Ino80 and overexpressing lys20-cat. Lanes 3–6 were from a strain expressing Ino80-myc and lys20-cat or lys20-moon from 2-μ plasmids. Note that compared to whole cell lysates in (E), IP samples of both myc-tagged Ino80 and Lys20 are proteolytically processed. (**E**) Ino80 protein levels were unaffected by Lys20 overexpression. Whole cell lysates of strains in (C) were probed with anti-Myc. (**F**) Histone H2A phosphorylation upon DNA damage was normal in *esa1* strains transformed with vector or overexpressing *LYS20*. Protein lysates of the indicated strains were probed with anti-phospho-H2A and anti-H2A antibodies after being treated with hydroxyurea (0.2 M for 90 min). Histone expression is reduced when cells are treated with hydroxyurea (64), thus the damage-induced increase in H2A phosphorylation is only apparent when the H2A phosphorylation signal is quantified relative to H2A levels. Approximately 2-fold induction was observed for all strains tested. The quantification was performed for two independent samples.

### Immunoprecipitations

Two hundred milliliter cultures were grown to A_600_ = 0.7. Pellets were lysed in 200 μl of cold IP lysis buffer by bead beating (50-mM HEPES-KOH pH = 7.5, 100-mM NaCl, 1-mM ethylenediaminetetraacetic acid (EDTA), 0.25% Nonidet-P40, 10% glycerol, 0.05% β-mercaptoethanol, 50-mM sodium butyrate, 50-mM NaF, 50-mM nicotinamide and a cocktail of protease inhibitors). The cleared lysate was incubated for 2 h with a 1:60 dilution of anti-Myc (9E10.2). The IP mixture was incubated for 1 h at 4°C with Protein A-Sepharose. Beads were washed three times with IP buffer and two times with wash buffer (50-mM HEPES-KOH pH = 7.5, 250-mM NaCl, 1-mM EDTA, 10-mM nicotinamide and 10-mM sodium butyrate).

## RESULTS

### A C-terminal moonlighting domain in Lys20

Lys20 was defined as a moonlighting protein because the *lys20-E155A* mutant was catalytically inactive as HCS, however it still suppressed the DNA damage sensitivity of *esa1* (26). Notably, nuclear localization of Lys20 is necessary for suppression of *esa1*, but not for lysine synthesis. As a first approach to characterize the mechanism of suppression we sought to identify the protein motif necessary for its role in DNA damage repair. Structure–function analyses were performed by mutating specific amino acids and domains of the protein. The mutant versions of Lys20 were expressed in two different strains to test for function in amino acid biosynthesis and in DNA damage repair. To analyze the biosynthetic role, we used a strain with deletions of *LYS20* and *LYS21*, which is a lysine auxotroph. Expression of catalytic-proficient *LYS20* alleles promoted growth in the absence of lysine. To investigate if the *LYS20* mutants retained activity in the DNA damage repair pathway, the *esa1–414* strain was transformed with the mutant versions of Lys20 and tested for sensitivity to DNA damage.

*LYS20* mutants tested (Figure 1A–C) included the H212, H214 residues that localize close to the catalytic E155 residue and contribute to metal binding. The G241A, G234A and the E244A, R245A mutants are present in an extremely conserved patch (GIGERNG) structurally localized between the catalytic (E155) and substrate binding residues (R31). Because Lys20 functions as a dimer (44), we also analyzed residues contributing to a lid that contacts the catalytic region of the second monomer: H309, I313, L314 and Y320 (Figure 1B). All amino acids tested were mutated to alanine.

Because Lys20 was a better suppressor of *esa1* than Lys21 (26), the C-terminal region of the protein, which differs in sequence between the isozymes, was also analyzed. A tract of 20 amino acids (V399-I418) preceding the nuclear localization signal (NLS) was deleted from Lys20 without affecting the NLS (Figure 1C).

The stability of the mutant proteins was analyzed by western blot, verifying that all mutants constructed were stable and expressed at similar levels (Supplementary Figure S1A). As nuclear localization was a requirement for suppression of *esa1*, the subcellular localization of the mutants was tested. All remained nuclear, including the C-terminal deletion mutant (Supplementary Figure S1B).

The analysis revealed that of the individual residues tested, I313 and L314 were dispensable for lysine biosynthesis (Figure 1D). Only G241 and G243 were necessary for both biosynthesis and suppression of *esa1* DNA damage sensitivity (Figure 1E). In contrast, the C-terminal patch of Lys20 was necessary to rescue *esa1* DNA damage sensitivity (Figure 1E), but not for lysine biosynthesis. Thus, we define the moonlighting domain of Lys20 to be localized within the 20 C-terminal amino acids preceding the NLS. For simplicity, the *lys20-V399-I418Δ* mutant that impairs the moonlighting role of Lys20 is denoted as *lys20-moon* hereafter.

To further dissect the 20 amino acid moonlighting domain of Lys20, a six amino acid deletion, containing four lysines (K403-N408), was constructed, along with alanine replacements of residues S401, S410 and E414 (Supplementary Figure S1C). The moonlighting function was somewhat compromised in the *K403-N408Δ* mutant; however, compared to wild-type *LYS20*, the other mutants tested were also slightly impaired in suppression of damage sensitivity. This suggested that multiple amino acids in the domain contributed to the moonlighting function of Lys20. Because *lys20-moon* was the only mutant tested that was completely impaired in the DNA damage repair role of Lys20 without losing the HCS activity, it was used for further analyses. The *lys20-E155A* mutant, hereafter referred to as *lys20-cat*, retained the moonlighting role in damage repair but lost the biosynthetic activity of Lys20, and is used below for comparison to *lys20-moon*.

### Lys20 genetically interacted with other DNA damage regulators

In addition to promoting DNA damage repair in *esa1* cells, Lys20 can also antagonize repair. This is revealed upon mutation of *LYS20*, which relieves the damage sensitivity of cells with deletion of the histone variant *HTZ1* and enhances Rad53 phosphorylation as part of the repair signaling cascade (26).

To further establish Lys20′s contributions to damage repair, we asked if it could suppress other mutant strains affecting distinct steps of the damage-signaling pathway. One of the initial steps in response to DNA DSBs includes phosphorylation of histone H2A at serine 129 by the ATR and ATM kinases, Mec1 and Tel1 (45). H2A phosphorylation spreads at both sides of the break and is a central binding site for repair factors (29). Other histone modifications that are important at the break include acetylation of the histone H4 tail (22) and replacement of the phosphorylated H2A with the histone variant Htz1 or H2A.Z (46,47). Histone H4 acetylation is established early after breaks occur (48) and is mediated by the HAT Esa1. Acetylation is later removed by deacetylases including Rpd3 and Sir2 (23,49).

Strains tested for suppression by *LYS20* included a histone H2A mutant that cannot be phosphorylated at serine 129 (H2A-S129A), a histone H4 tail deletion and an *HTZ1* null strain. Proteins involved in regulation of chromatin structure during damage were tested, and included an *SDS3* null (lacking the Rpd3L deacetylase complex) and a *RAD6* null (which encodes an ubiquitin conjugating enzyme). A *tel1Δ* strain was tested as a mutant affecting kinase signaling, and *rad52Δ* as loss of a late effector during homologous recombination (HR) repair (50). In the mutants studied, Lys20 overexpression was neutral, or acted in two ways: it robustly suppressed the damage sensitivity of *esa1–414* and more mildly suppressed *tel1Δ.* It exacerbated damage in the *rad6Δ* strain and had no effects on the other mutants tested (Figure 2). Thus, Lys20 can act divergently in DNA damage repair, with both positive and negative effects. As Lys20 overexpression best suppressed *esa1*, we further characterized this genetic interaction.

### Lys20 was recruited to DSB with similar kinetics as Esa1

*ESA1* mutants have reduced global histone H4 acetylation levels that correlate with altered gene expression upon DNA damage (A. L. Torres-Machorro, L. G. Clark *et al.*, submitted). As Esa1 is recruited directly to DSBs as part of the NuA4 complex (22,48), it is presumed, although not yet demonstrated, that *esa1* strains have defects in histone acetylation at breaks that result in impaired damage signaling. The mechanism of suppression of *esa1* by Lys20 overexpression could relate to increased histone acetylation, perhaps improving the transcriptional response upon damage. Suppression of *esa1*′*s* DNA damage sensitivity has been observed when the Set3 deacetylase complex is removed, leading to a global increase in histone H4 acetylation (A. L. Torres-Machorro, L. G. Clark *et al.*, submitted). However, when *LYS20* was overexpressed in *esa1* mutants, we did not observe changes in global H4 acetylation (Figure 3A). For this reason we decided to analyze the possibility of a direct role of Lys20 at DNA DSBs.

Lys20 is a predominantly nuclear protein that is not freely diffusible (11). To test whether Lys20 directly bound to chromatin, subcellular fractionation to enrich for chromatin was performed (37). Controls for the fractionation were a known chromatin bound protein, the deacetylase Sir2, and a predominantly cytoplasmic protein, the Pgk1 glycolytic enzyme. As expected, Sir2 was enriched in the crude chromatin fraction (Crude Chr) and in the purified chromatin fraction (Chr), whereas Pgk1 was enriched in the soluble (Sol) and high-speed supernatant (Hs) fractions. Figure 3B shows that Lys20 was abundant in the soluble fraction. To stabilize interactions, we repeated the fractionation adding a ChIP-like crosslinking step before the procedure. Fractionations were cleaner when crosslinking was used, with enrichment of Sir2 in the chromatin fractions, and Pgk1 in the soluble fractions. With these conditions, Lys20 was now enriched in the crude chromatin and chromatin fractions, suggesting that Lys20′s interaction with chromatin is transient or unstable. We also found that upon DNA damage induction, more Lys20 bound to chromatin compared to non-induced conditions. This result was also seen with Sir2, which has been previously linked to DNA damage repair and found to be localized to sites of damage (23).

The fractionation results prompted us to assess binding of Lys20 to DNA DSBs. We took advantage of the well-characterized approach of galactose-mediated induction of the HO endonuclease to create a single genomic DSB (41). This enzyme normally cuts at a single site within the mating-type locus, resulting in repair by HR with the silent mating-type loci (51). Growth in galactose induces HO to yield a DSB in 90% of cells after 1 h (23,29). Cells were grown in galactose for one or more hours, and then ChIP was used to evaluate the kinetics of protein recruitment after damage.

As *LYS20* was found to interact genetically with *ESA1*, and Esa1 is recruited to the breaks with known kinetics (23), we used Esa1 as a control. Recruitment of both proteins 0.2 kb downstream of the break was analyzed 1, 2 and 3 h after HO induction. A 2-fold enrichment of Esa1 was previously found at the 2-h time point. We reproduced the kinetics for the Esa1 protein (Figure 3C) and found that the truncated esa1–414 protein was recruited with very similar kinetics to wild-type Esa1 (Supplementary Figure S2A). Lys20 was also found enriched 3 h after break induction (Figure 3D). The recruitment of Lys20 was unaffected by the presence of the partially inactive esa1–414 enzyme in comparison to wild-type Esa1 (Supplementary Figure S2B). Thus, Lys20 is normally recruited to the DSBs with similar kinetics to Esa1 and esa1–414.

As overexpression of Lys20 suppressed the DNA damage sensitivity of *esa1–414*, one straightforward hypothesis is that increased expression of Lys20 could lead to its increased accumulation at the breaks that could promote higher levels of recruitment of esa1–414. Lys20 overexpression did lead to higher Lys20 levels at the break compared to a vector transformed strain (Figure 3D). Lys20 was also recruited earlier and continued to accumulate at the breaks to reach almost twice the amount of protein found with endogenous levels of expression. However, in contrast to the recruitment hypothesis, we found that esa1–414 levels were similar in the Lys20 overexpression strain compared to the vector transformant (Figure 3C). Thus, in agreement with the lack of physical interaction between Esa1 and Lys20 (26), increased recruitment of Esa1 to the breaks was not a mechanism of suppression by Lys20.

The results above were tested in a strain lacking the silent mating-type loci, meaning that no homology existed to promote repair by HR. Thus, in galactose, the DSB persisted and allowed analyses of protein recruitment in the absence of repair by HR. However, increased histone acetylation at breaks is more readily detected when cells can undergo repair (23). Because we also wanted to analyze histone acetylation and the behavior of the proteins during repair, we tested recruitment of Lys20 and Esa1 in a strain with intact *HM* silent mating-type loci that was thus repair competent. Results with these repairing strains were similar to the non-repairing strains. Esa1 was recruited after 1 h of break induction and had increased levels 1 h later. After HO was repressed by glucose and cells were allowed to initiate repair, Esa1 was lost from the break (Figure 3E). Lys20 was also recruited at high levels during the first 2 h of HO induction (Figure 3F), however during repair, although its levels were reduced, it remained at the break.

Esa1 was previously reported to be recruited close to the DSB but not found enriched at greater distances from the break (e.g. 2 kb). We found that this was also the case for Lys20, which was not enriched 2 kb downstream from the break (Supplementary Figure S2C). Thus, Lys20 and Esa1 are recruited to the breaks with similar localization and kinetics. However, when Lys20 is overexpressed, higher levels of Lys20 are recruited and it remains at the breaks during repair for greater duration compared to Esa1.

### Lys20 overexpression suppressed the histone eviction defect of *esa1* at DSBs

H4K5 and K16 acetylation are normally increased 1 and 2 h after DSB induction (23) (Figure 4A–C). During the same time, histones are also depleted from the break (Figure 4B) to allow recruitment of factors involved in later steps of the repair process (32).

We asked if acetylation marks at the HO-induced break were affected in *esa1* cells and whether recruitment of Lys20 would suppress the defect, if present. The wild-type strain showed a normal increase in H4K5 acetylation 1 and 2 h after break induction, whereas *esa1* cells had no clear increase in H4 acetylation at the same time points (Figure 4A). We analyzed histone H4 acetylation levels in *esa1* strains transformed with vector, with *lys20-cat* and with *lys20-moon*. Compared to the unchanged acetylation in vector-transformed *esa1* cells, H4K5 acetylation was elevated when *lys20-cat* was overexpressed (Figure 4D). By contrast, increased H4K5 acetylation was delayed in the *esa1* strain transformed with *lys20-moon* (Figure 4D). Histone H3 levels were also measured at the break for all conditions. Strikingly, instead of being depleted as in wild-type cells, H3 persisted at the break in *esa1* cells (Figure 4B). Vector and *lys20-moon* transformed *esa1* strains failed to evict histone H3 (Figure 4E). Conversely, when *lys20-cat* was overexpressed, histones were normally depleted from the breaks (Figure 4E). When histone H4K5 acetylation was calculated relative to histone levels (52), an increase in acetylation similar to wild-type (Figure 4C) was only observed when the moonlighting-proficient protein lys20-cat was overexpressed in *esa1* mutant cells (Figure 4F). After repair had started, the kinetics were similar to previous data (23), where histone acetylation was reduced.

Acetylation of H4K16 was analyzed in a similar manner. In this case, a comparable increase in H4K16 acetylation was found 1 h after break induction when both *lys20-cat* and *lys20-moon* were overexpressed (Figure 4G). Nevertheless, when H4K16 acetylation relative to histone levels was calculated, only lys20-cat showed a normal increase in H4 acetylation, whereas the strain overexpressing the repair-deficient lys20-moon had low relative levels of H4K16 acetylation (Figure 4H).

As the moonlighting defective mutant was unable to promote histone loss at the breaks, we asked if this result was due to lys20-moon recruitment to the breaks being impaired. Figure 4I shows that lys20-cat was normally recruited to the DSB, having the same kinetics at the break as wild-type Lys20 (Figure 3D). Conversely, the recruitment of lys20-moon to the breaks was reduced and delayed relative to lys20-cat (Figure 4I), suggesting that the C-terminal domain of Lys20 plays an important role in the recruitment of Lys20 to the breaks.

### Lys20 promoted elevated Ino80 levels at the break

The results above demonstrated that the *esa1* strain had impaired signaling at DSBs, characterized by compromised histone eviction and loss of acetylation early during the repair process. Overexpression of the metabolic mutant lys20-cat suppressed this defect by promoting timely histone depletion and higher relative levels of acetylation in response to the DSBs. This led us to speculate that *esa1* cells were impaired for remodeling at the breaks. Chromatin remodelers are important components of the repair pathway that work at different steps of the signaling at the sites of DNA damage (53,54). The RSC complex directs nucleosome sliding shortly after DSB formation (55,56,57) and also has a later role following synapsis for completion of the recombinational repair (58). The INO80 complex removes histones early during repair, after being recruited by phosphorylated H2A (32,35). The SWI/SNF complex is linked to later steps of the repair pathway, prior to synapsis, and functions at the donor sequence to expose DNA to homology search (58).

To ask if suppression by Lys20 was dependent on a specific remodeling complex, we constructed mutants affecting the RSC, INO80 or SWI/SNF complexes in the *esa1–414* strain. Deletion of the *ARP8* subunit of the INO80 complex results in compromised ATPase activity, DNA binding and nucleosome mobilization by INO80 (28). To impair the nucleosome sliding role of RSC (59), the *RSC1* subunit was deleted, whereas the activity and assembly of the SWI/SNF complex were impaired by deletion of the *SNF5* subunit (60).

Figure 5A shows that suppression of *esa1–414* by Lys20 overexpression was independent of the RSC and SWI/SNF complexes, however it was dependent on the INO80 complex as the DNA damage sensitivity of *esa1–414 arp8Δ* was not suppressed by Lys20 overexpression. Because Ino80 is recruited to HO-induced breaks with similar localization and kinetics as the NuA4 complex (32,48), we proceeded to analyze Ino80. We found that in *esa1* cells recruitment of Ino80 was similar to wild-type (Figure 5B). However, when *lys20-cat* was overexpressed, the recruitment of Ino80 was enhanced 1 h after break induction, reaching a 12-fold increase relative to time zero (Figure 5C). Finally, the repair-deficient *lys20-moon* showed a similar pattern to the vector-only strain. However, a small increase in Ino80 recruitment to the break was observed 2 h after break induction. This result correlated with some level of recruitment of lys20-moon that was low compared to lys20-cat (Figure 4I) 2 h after break induction.

To asses if effects on Ino80 recruitment could be mediated through interaction between Lys20 and Ino80, we performed IPs in a strain expressing myc-tagged Ino80 and lys20-cat. The anti-Myc blot in Figure 5D demonstrated efficient pull-down of the Ino80 protein. Co-IP was tested by probing the same membrane with anti-Lys20 antibody. No signal was observed with untagged Ino80, whereas lys20-cat was co-immunoprecipitated with Ino80-myc, suggesting physical interaction between Lys20 and Ino80. Because whole cell lysates were used for the IPs, the interaction may be direct or it may be indirect, resulting from bridging with other interacting proteins.

Comparative sequence analysis of the 20 amino acids of the moonlighting domain revealed similarity to non-catalytic domains of mammalian homologs of slingshot phosphatases. The region of homology in these phosphatases (61) is predicted to be unstructured (62) and can be important for interaction with actin (63). We asked if the moonlighting domain was important for the interaction with Ino80, perhaps through actin or actin-related proteins found in the INO80 complex (27). We found that the moonlighting domain was not necessary for interaction with Ino80, as the lys20-moon mutant lacking the domain also co-precipitated with Ino80 (Figure 5D). The finding that lys20-cat and lys20-moon have similar degrees of interaction with Ino80 correlates with DSB recruitment of Lys20 and Ino80: lys20-cat recruitment promoted enrichment of Ino80 at the breaks, whereas lys20-moon was poorly recruited to the breaks. Protein levels of lys20-moon at the DSB trended similarly to the low Ino80 enrichment observed at the same time points.

Ino80 protein levels were unaffected by overexpression of *LYS20* mutants (Figure 5E), suggesting that Lys20 overexpression promoted increased recruitment rather than increased expression of Ino80 in *esa1* cells. As DSB recruitment of various repair factors is dependent on H2A phosphorylation (22,29), we analyzed global H2A phosphorylation in *esa1* cells transformed with vector or overexpressing *LYS20* mutants. The induction of H2A phosphorylation upon damage (64) was comparable in wild type and *esa1* strains, either transformed with vector or with Lys20 (Figure 5F). This result suggested that *esa1* or *LYS20* overexpressing cells are not defective in H2A signaling. We also found that H2A phosphorylation was not important for Lys20 recruitment to the breaks (Supplementary Figure S3).

**Figure 6.**
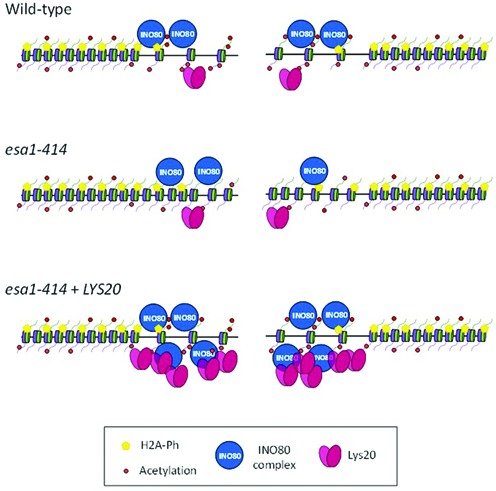
*LYS20* overexpression suppressed *esa1* histone eviction defect at the breaks. Cartoon representing a simplified chromatin landscape in wild-type, *esa1* and *esa1*+*LYS20* strains. The impaired histone acetylation (red balls) and histone eviction at the DSBs in *esa1* cells were rescued by *LYS20* overexpression through increased recruitment of INO80 (blue circles). Histone H2A phosphorylation (yellow pentagons) was unaffected.

Overall, *esa1–414* mutants have defective acetylation and histone eviction at the DSBs. These defects are suppressed by the moonlighting function of Lys20. Lys20 is normally recruited to DSBs, particularly robustly when it is overexpressed. High Lys20 levels at the break promote increased recruitment of the INO80 remodeling complex that correlate with normal histone eviction and suppression of *esa1* DNA damage sensitivity (Figure 6).

## DISCUSSION

Few bifunctional proteins with metabolic roles have been defined for their functions in chromatin. Thus, Lys20′s characterization as a bifunctional protein is significant for understanding new aspects of protein evolution and response to DNA damage. First we localized the moonlighting domain of the protein to the C-terminal region of Lys20, an area structurally independent of the catalytic domain.

The physicochemical properties of the moonlighting domain, although not the specific sequence, are found in the C-terminal region of HCS of other fungi, including some Cryptococcal pathogens. Such sequence divergence is a recognized property among multiple moonlighting domains and is considered to be evidence for recent evolution of these additional functions (4). The presence of moonlighting HCSs in select fungi may provide a fitness advantage under stress conditions as discussed below.

The moonlighting domain of Lys20 has a theoretical isoelectric point of 8.4 and is predicted to be intrinsically unstructured (62). This characteristic has been found in other moonlighting proteins, potentially conferring flexibility to interact with different protein or protein–nucleic acid complexes (3,65). We found that the moonlighting domain of Lys20 is important for recruitment to sites of DNA damage but not for interaction with the Ino80 complex. As H2A phosphorylation was not necessary for Lys20 binding to the DSB, it remains to be established whether its recruitment and interaction with Ino80 are direct or mediated by another factor or chromatin signal.

*LYS21*, the paralog of *LYS20*, is a poor suppressor of *esa1* compared to Lys20 (26). The C-terminal region of Lys21 does not include the moonlighting domain that we defined here for Lys20. We propose that the absence of the moonlighting domain in Lys21 explains its compromised function in DNA damage repair.

We characterized the mechanism of suppression of the DNA damage sensitivity of mutant *esa1* cells by the catalytically inactive yet moonlighting-competent variant of the metabolic protein Lys20. As overexpression of Lys20 did not improve the low global acetylation of H4 in *esa1* cells, we sought to define the mechanism of its suppression. We initially characterized the chromatin landscape of *esa1–414* cells at a single induced break. As expected, low levels of histone H4 acetylation were observed close to the break. Unexpectedly however, we also found lack of histone eviction in *esa1* mutants, suggesting impaired chromatin remodeling (Figure 6). This is in agreement with a previous report that found reduced levels of recruitment of the INO80 subunit Rvb1 at DSBs in *esa1* cells (48). Under suppressing conditions, when *lys20-cat* was overexpressed in *esa1* cells, histone H4K5 acetylation was increased 1 h after break induction, an increase that may be mediated by the non-canonical HAT activity of Lys20 (26). The result was clearer, however, when histone acetylation levels were analyzed relative to the total histone levels, which were only low when *lys20-cat* was overexpressed and not when *lys20-moon* or vector was transformed. This result suggested that the defect in *esa1* cells was a lack of histone eviction in response to DSBs.

We observed that suppression by Lys20 was dependent on the INO80 complex. Lys20 promoted an increase in Ino80 accumulation at the break that correlated with normal histone eviction in response to damage (Figure 6). Ino80 recruitment appears to be mediated through interaction with Lys20. An additional possibility is that recognition of increased histone acetylation at the breaks could also signal INO80 recruitment, as this mark was previously shown to be important for normal recruitment of the complex (48).

There is a central role for INO80 in *LYS20*′s contribution to damage repair. Not only does *LYS20* fail to suppress in the *esa1 arp8Δ* mutant, the DNA damage sensitivity of the *ARP8* null strain itself was worsened by overexpression of *LYS20* (Figure 5). As part of the INO80 complex remains intact in *arp8Δ* cells, we hypothesize that in this compromised state, Lys20 overexpression promotes high levels of recruitment of inactive INO80 to the breaks. Such inoperative complexes would be unable to promote histone eviction but could instead block recruitment of other remodeling complexes and repair proteins, resulting in the worsened damage sensitivity seen in *arp8Δ* cells.

*LYS20* null cells are somewhat resistant to DNA damage, probably through increased Rad53 checkpoint signaling (26), whereas high levels of expression also have a role in DNA damage repair, as shown here and in the earlier study (26). Thus, like other proteins active in epigenetic processes, Lys20 is likely to function in repair through more than one mechanism. We propose then that Lys20 contributes to damage repair via a mechanism that is put into place when other pathways for repair are impaired, such as the compromised histone H4 acetylation at breaks in *esa1* mutants. In this regard, Lys20 joins the ranks of other proteins with backup roles in repair, such as the human Poly (ADP ribose) polymerase 1 (Parp-1) and ligase Lig1 (66); and the AlkB dioxygenase of *Pseudomonas putida* (67).

Even though Lys20 protein levels are not strongly affected by DNA damage (Supplementary Figure S4), physiological conditions can increase its expression, for example when there is no lysine in the medium (Supplementary Figure S4). In fact, Lys20 protein levels are regulated by environmental conditions, whereas the protein levels of its isozyme Lys21 remain constant (68) (Supplementary Figure S4). Among others, these conditions include amino acid availability (11), carbon source (68), levels of the reactive oxygen species superoxide and growth at different cell densities (Supplementary Figure S4). The regulation of Lys20 protein levels by the environment suggests that its moonlighting function may be similarly regulated.

In summary, we found that the moonlighting domain of Lys20 is localized to the C-terminus of the protein. This domain is important for recruitment of Lys20 to DNA double-strand breaks, where it promotes increased levels of recruitment of the INO80 complex. The dynamic contribution of an amino acid biosynthetic protein to DNA damage repair underscores the intertwined evolution of metabolic and chromatin mediated processes.

## SUPPLEMENTARY DATA

Supplementary Data are available at NAR Online.

SUPPLEMENTARY DATA
