# Supplementary material for: A moonlighting metabolic protein influences repair at DNA double-stranded breaks
Source: Nucleic Acids Res. 2015 Jan 27;43(3):1646–58. doi: 10.1093/nar/gku1405 (PMC4330366; doi:10.1093/nar/gku1405)
Supplement: SUPPLEMENTARY DATA [file supp_43_3_1646__index.html]

A moonlighting metabolic protein influences repair at DNA double-stranded breaks — SUPPLEMENTARY DATA 

# A moonlighting metabolic protein influences repair at DNA double-stranded breaks

## SUPPLEMENTARY DATA

**Files in this Data Supplement:**

- SUPPLEMENTARY DATA
