# Supplementary material for: A moonlighting metabolic protein influences repair at DNA double-stranded breaks
Source: Nucleic Acids Res. 2015 Jan 27;43(3):1646–58. doi: 10.1093/nar/gku1405 (PMC4330366; doi:10.1093/nar/gku1405)
Supplement: SUPPLEMENTARY DATA [file supp_gku1405_nar-02575-d-2014-File008.pdf]

A moonlighting metabolic protein influences repair at DNA double-stranded breaks

Ana Lilia Torres-Machorro, John P. Aris and Lorraine Pillus

Table S1. Yeast strains

Strain	Genotype	Reference
LPY5	*MATa W303 (ade2-1 can1-100 his3-11 leu2-3,112 trp1-1 ura3-1)*	
LPY79	*MATα W303*	
LPY3291	*MATa S288c esa1Δ::HIS3 + pLP863*	
LPY3486	*MATa S288c (his3Δ200 leu2-3,112 trp1Δ1 ura3-52)*	
LPY4774	*MATa W303 esa1-414*	
LPY4776	*MATα W303 esa1-414*	
LPY6282	*MATα W303 rDNA::ADE2-CAN1*	
LPY6491	*MATα BY (his3Δ1 leu2Δ0 met15Δ0 ura3Δ0)*	
LPY11412	*MATα W303 rDNA::ADE2-CAN1 lys20Δ::KanMX lys21Δ::natR*	
LPY12830	*MATα W303 htz1Δ::KanMX*	
LPY12959	*MATα W303 sds3Δ::KanMX*	
LPY14264	*MATα ade1 leu2-3,112 lys5 trp1::hisG ura3-52 hml::ADE1 hmr::ADE1 ade3::Gal-HO (JKM179)*	J. Haber
LPY15845	*JKM179 esa1-414-13MYC-KanMX*	
LPY15904	*JKM179 ESA1-13MYC::KanMX*	
LPY16465	*MATa ade1 leu2-3,112 lys5 trp1::his5 ura3-52 hml::ADE1 hmr::ADE1 ade3::Gal-HO (JKM139)*	J. Haber
LPY16974	*MATa W303 ade3::Gal10::HO esa1-414-13MYC-KanMX*	
LPY17213	*MATa W303 ade3::Gal10::HO (BAT009)*	23
LPY17707	*MATa W303 ade3::Gal10::HO lys20Δ::KanMx lys21Δ::natR*	
LPY17709	*MATα W303 ade3::Gal10::HO lys20Δ::KanMx lys21Δ::natR*	
LPY18023	*MATα W303 ade3::Gal10::HO esa1-414-13MYC-KanMX*	
LPY18397	*MATα W303 ade3::Gal10::HO esa1-414*	
LPY18401	*MATα W303 ade3::Gal10::HO esa1-414 lys20Δ::KanMX lys21Δ::natR*	
LPY19108	*MATα W303 ade3::Gal10::HO*	
LPY19357	*MATα W303 rad6Δ::KanMX*	
LPY19617	*MATα W303 rad52::LEU2*	
LPY19629	*MATα W303 hht1-hhf1Δ::KanMX hta1-htb1Δ::NAT hta2-htab2Δ::HPH + pLP2863*	
LPY19631	*MATα W303 hht1-hhf1Δ::KanMX hht2-hhf2Δ::KanMX hta2-htb2Δ::HPH + pLP1492*	
LPY19782	*MATα W303 tel1Δ::KanMX*	
LPY19824	*MATα W303 arp8Δ::KanMX*	
LPY19828	*MATα W303 arp8Δ::KanMX esa1-414*	
LPY20071	*MATα W303 rsc1Δ::KanMX*	
LPY20072	*MATα W303 rsc1Δ::KanMX esa1-414*	
LPY20075	*MATα snf5Δ::kanMX*	
LPY20076	*MATα W303 snf5Δ::KanMX esa1-414*	
LPY20113	*MATα JKM179 INO80-13MYC-TRP1 HTB1-FLAG-LEU2 (MAO104)*	32
LPY20339	*MATα W303 ade3::Gal10::HO esa1-414 INO80 13MYC::TRP1*	
LPY20531	*MATα W303 ade3::Gal10::HO INO80 13MYC::TRP1*	
LPY20539	*MATα W303 ade3::Gal10::HO esa1-414 lys20Δ::KanMX lys21Δ::natR INO80 13MYC::TRP1*	
LPY21643	*MATα W303 ade3::Gal10::HO hht1-hhf1Δ::KanMX hta1-htb1Δ::NAT hta2-htab2Δ::HPH + pLP3308*	
LPY21644	*MATα W303 ade3::Gal10::HO hht1-hhf1Δ::KanMX hta1-htb1Δ::NAT hta2-htab2Δ::HPH + pLP2863*	

Table S2. Plasmids

Strain	Details	Reference
pLP796	*ESA1 in URA3 2μ*	
pLP863	*esa1-414 in TRP1 CEN*	
pLP1402	*pRS202 (URA3, 2μ)*	
pLP1412	*LYS20 in PRS202*	
pLP1455	*pFA6a-ESA1-13MYC-KanMX6-ESA1-3'*	
pLP1492	*H3 + H4-NtailΔ in TRP1, CEN*	*
pLP2365	*lys20-E155A in pRS202*	
pLP2558	*lys20-K422N in pRS202*	
pLP2564	*pFA6a-esa1-414-13MYC-KanMX6-ESA1-3'*	
pLP2580	*lys20-H309A in pRS202*	
pLP2581	*lys20-V399-I418Δ in pRS202*	
pLP2582	*lys20-H212A, H214A in pRS202*	
pLP2605	*lys20-E244A, R245A in pRS202*	
pLP2606	*lys20-G241A, G234A in pRS202*	
pLP2607	*lys20-I313A, L314A in pRS202*	
pLP2608	*lys20-Y320A in pRS202*	
pLP2731	*lys20-S410A in pRS202*	
pLP2732	*lys20-S401A in pRS202*	
pLP2733	*lys20-K403-N408Δ in pRS202*	
pLP2734	*lys20-E414A in pRS202*	
pLP2863	*H2A-S129A + H2B in TRP1, CEN*	**
pLP3308	*H2A + FLAG-H2B in TRP1, CEN*	***

* Zhang, W. *et al*., (1998) Essential and redundant functions of histone acetylation revealed by mutation of target lysines and loss of the Gcn5p acetyltransferase. *EMBO J*. **17**:3155-67.
** Nakanishi, S., et al., (2008) A comprehensive library of histone mutants identifies nucleosomal residues required for H3K4 methylation. *Nat. Struct. Mol. Biol.* **15**:881-8.
*** Basnet H., et al., (2014) Tyrosine phosphorylation of histone H2A by CK2 regulates transcriptional elongation. *Nature* **516**:268-271.

Table S3. Oligonucleotides

Number	Name	Sequence	Reference
oLP1275	SCR1-F	CGCGGCTAGACACGGATT	*
oLP1276	SCR1-R	GCACGGTGCGGAATAGAGAA	*
oLP1487	MUTL20-K422N-F	CATCCCCGCCGCAATCGGACTAAGCCATCC	
oLP1488	MUTL20-K422N-R	GGATGGCTTAGTCCGATTGGCGGCGGGGATG	
oLP1493	ESA1-1240D-F	GATATTTTAGATAGGTACATCGACTTAAAGCC	
oLP1494	ESA1-1240D-R	GGCTTTAAGTCGATGTACCTATCTAAAATATC	
oLP1500	ESA1-LIN-F	GCCAAAAAGAGAAGGACAAGGGATCCCCGGGTTAATTAAC	
oLP1501	ESA1-LIN-R	GTTAATTAACCCGGGGATCCCTTGTCCTTCTCTTTTTGGC	
oLP1508	MAT-HO-F	GGAGGTTGTTTATCTTTCGAGTACTGA	B. Braunstein
oLP1509	MAT-HO-R	TTGATTGTTTGCTTGAGTCTGAGTAAT	
oLP1510	LYS20-H212A-F	CATGTGACATCGAATGCGCTTTCGCCAACGATACTGGTTG	
oLP1511	LYS20-H212A-R	CAACCAGTATCGTTGGCGAAAGCGCATTCGATGTCACATG	

oLP1512	LYS20-H309A-F	CATAAAGCAGGTATCGCTGCCAAGGCCATTTTG	
oLP1513	LYS20-H309A-F	CAAAATGGCCTTGGCAGCGATACCTGCTTTATG	
oLP1514	LYS20-V399-I418Δ-F	CAGAGGTCAGCACTCCTCAACCCGCCGCCAAGCGGACTAAG	
oLP1515	LYS20-V399-I418Δ-R	CTTAGTCCGCTTGGCGGCGGGTTGAGGAGTGCTGACCTCTG	
oLP1568	LYS20-E244AR245A-F	GTACTGGGTATTGGTGCAGCAAACGGTATCACTCC	
oLP1569	LYS20-E244AR245A-R	GGAGTGATACCGTTTGCTGCACCAATACCCAGTAC	
oLP1570	LYS20-G241AG243A-F	CGTCAGTGTACTGGCTATTGCTGAAAGAAACGGTATC	
oLP1571	LYS20-G241AG243A-R	GATACCGTTTCTTTCAGCAATAGCCAGTACACTGACG	
oLP1572	LYS20-I313AL314A-F	CCATGCCAAGGCCGCTGCGGCTAACCCATCTAC	
oLP1573	LYS20-I313AL314A-R	GTAGATGGGTTAGCCGCAGCGGCCTTGGCATGG	
oLP1574	LYS20-Y320A-F	GCTAACCCATCTACCGCCGAAATCTTGGACCC	
oLP1575	LYS20-Y320A-R	GGGTCCAAGATTTCGGCGGTAGATGGGTTAGC	
oLP1695	LYS20-K403N408Δ-F	CTCAAGTACTATCTGCAGACAGCGATGTACCG	
oLP1696	LYS20-K403N408Δ-R	CGGTACATCGCTGTCTGCAGATAGTACTTGAG	
oLP1697	LYS20-S401A-F	CACTCCTCAAGTACTAGCTGCAAAAAAGAACAAG	
oLP1698	LYS20-S401A-R	CTTGTTCTTTTTTGCAGCTAGTACTTGAGGAGTG	
oLP1699	LYS20-S410A-F	GAACAAGAAGAATGACGCCGATGTACCGGAAC	
oLP1700	LYS20-S410A-R	GTTCCGGTACATCGGCGTCATTCTTCTTGTTC	
oLP1701	LYS20-E414A-F	CAGCGATGTACCGGCACTGGCCACCATCCC	
oLP1702	LYS20-E414A-R	GGGATGGTGGCCAGTGCCGGTACATCGCTG	
oLP2035	INO80-ORF-F	CACAGGACTATTCCCTGAACC	
oLP2036	INO80-KO-R	CCGCGGAGGAAGCCAGTC	
Probe	SMC2	[6-FAM]ACGCCATTTGCTTCGTGCTCGGTA[TAMRA-6-FAM]	23
Probe	0.6KB HO	[5-HEX]ACTGCCCATGCGGTTCACATGACTT[TAMRA-5HEX]	23
Probe	2KB HO	[5-HEX]TCATCGAGCCCGTGAAGCATTCG[TAMRA-5HEX]	23
	P-SMC2-F	GGTCCGGTAAGTCGAACATTTT	23
	P-SMC2-R	CTCGCACAGTGCTCATTGATG	23
	P-0.6HO-F	TTGGATCTTAACAAACCGTAAAGGT	23
	P-0.6HO-R	GGTAACTAGCAAACAAAGGAAAGTCA	23
	P-2HO-F	CCATCGTGTTCATGGATCCTT	23
	P-2HO-R	AGAACATCCAGATTTGAACCGAA	23

* Yang, B. and Kirchmaier, A.L. (2006) Bypassing the catalytic activity of *SIR2* for SIR protein spreading in *Saccharomyces cerevisiae*. *Mol. Biol. Cell* **17**: 5287-97.

SUPPLEMENTARY METHODS

Immunostaining

As in (26) with the following modifications: the anti-Lys20 dilution was 1:500 and the dilution for the secondary antibody, Texas red-conjugated AffiniPure goat anti-mouse IgG, was 1:100 (Jackson ImmunoResearch Laboratories). The secondary antibody was incubated for 1h at room temperature and the cells were DAPI-stained for 2 minutes. An Axiovert 200M (Carl Zeiss MicroImaging) with a 100 × 1.3 NA objective was used for visualization of the cells. Images were captured with a monochrome Axiocam digital camera (Carl Zeiss MicroImaging) and analyzed using Axiovision software (Carl Zeiss MicroImaging).

SUPPLEMENTARY FIGURES

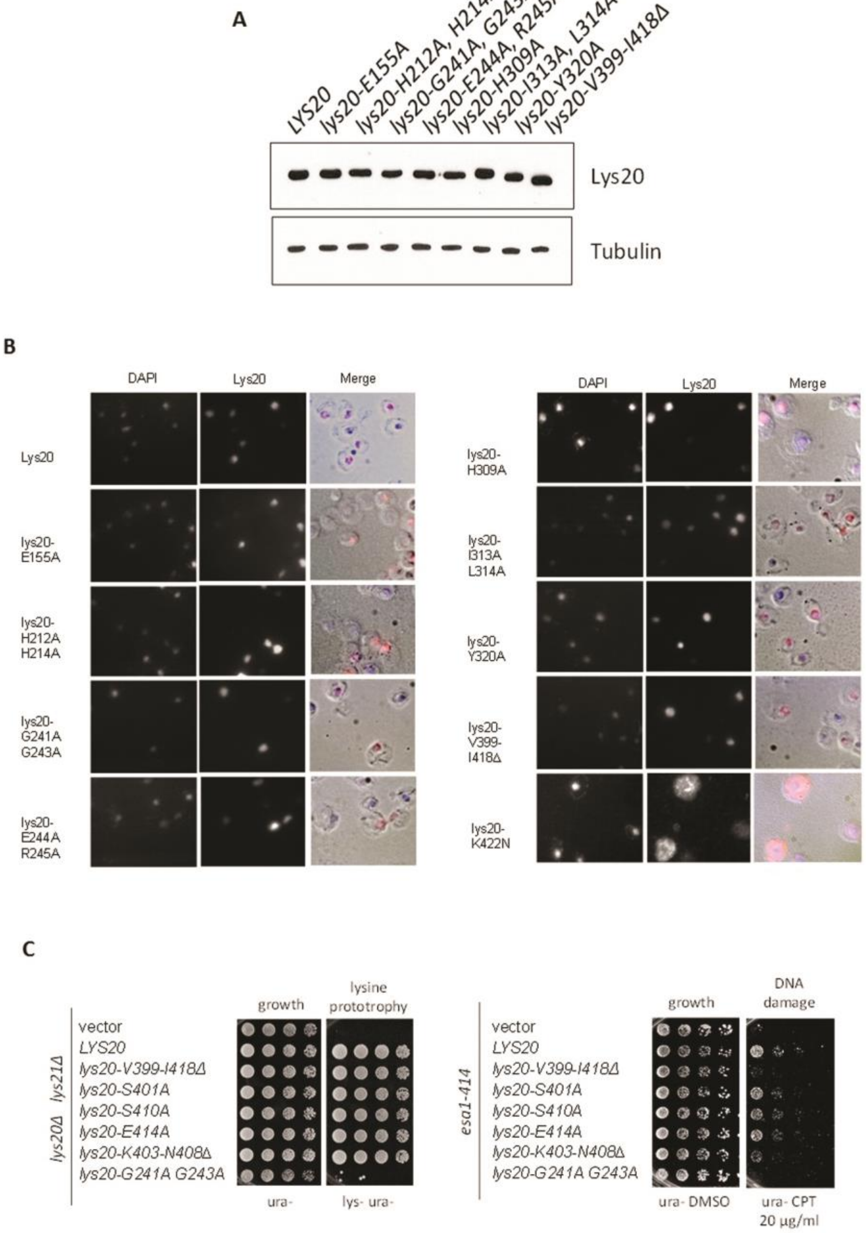

Figure S1. Mutant lys20 proteins had similar levels of expression and remained predominantly nuclear.

(A) The lys20 mutants had similar levels of expression. The mutants were expressed in a *LYS20 LYS21* null strain and grown to log phase. Protein lysates were probed for Lys20 and tubulin as a loading control. Note that the *lys20-V399-I418* (*lys20-moon*) mutant had slightly increased mobility due to loss of 20 amino acids. **(B)** Lys20 mutants were nuclear. The cellular localization of Lys20 mutants in (A) was evaluated by immunofluorescence. The mutants remained nuclear with the exception of the *lys20-K422N* control with a mutant NLS. **(C)** Further dissection of the moonlighting domain of Lys20. The mutants indicated were tested for their ability to synthesize lysine or suppress *esa1* in the same assays as those tested in Figure 1D-E.

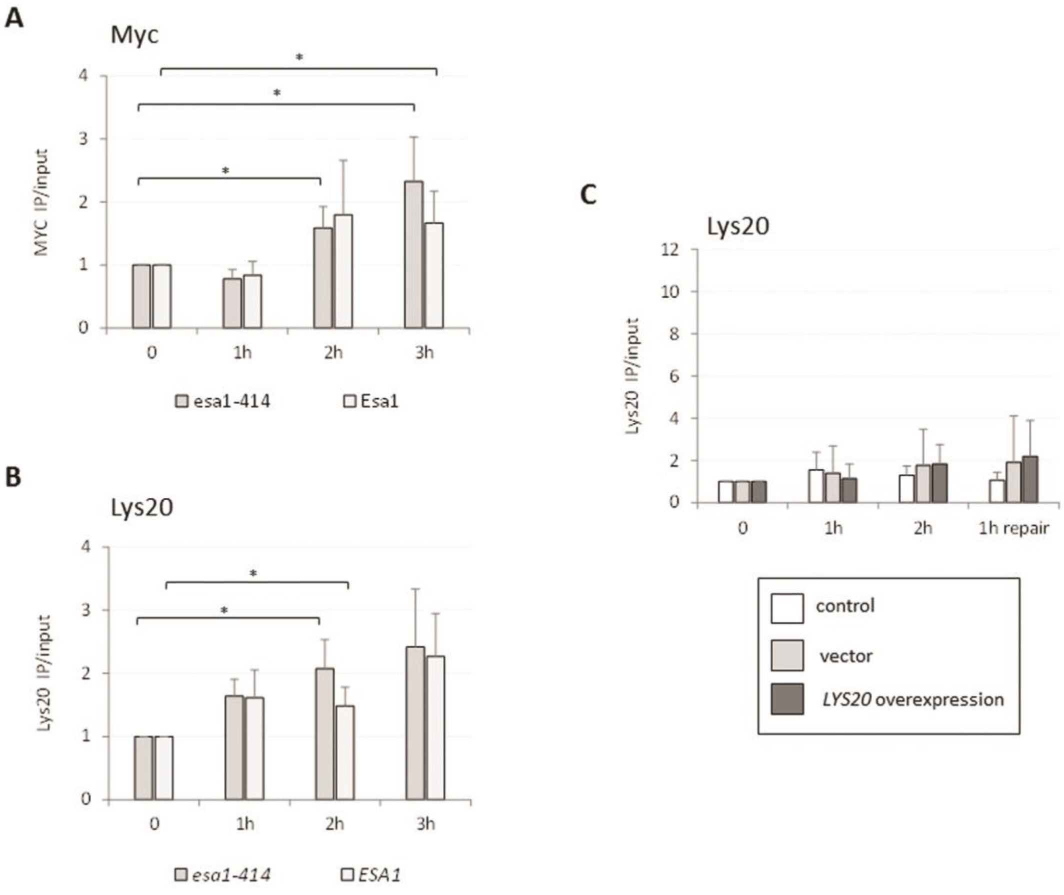

Figure S2. Control ChIP experiments.

(A) Esa1 and esa1-414 had similar kinetics of recruitment to the DSB. Myc-tagged Esa1 and Esa1-414 cells were used to perform the anti-Myc ChIP before and during HO induction. **(B)** Lys20 recruitment was similar in the presence of wild-type *ESA1* or *esa1-414*. The anti-Lys20 ChIP was performed in the strains tested in (A). **(C)** Lys20 was not recruited 2kb downstream of the break. The ChIPs in Figure 3F were tested here with probes and primers located 2kb downstream of the break.

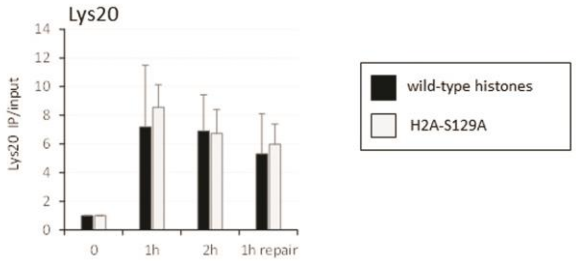

Figure S3. Lys20 recruitment to the DSB was not dependent on H2A-S129 phosphorylation.

Strains LPY21643 and 21644 were transformed with *lys20-cat*. Lys20 recruitment to the DSB was tested as in Figure 3.

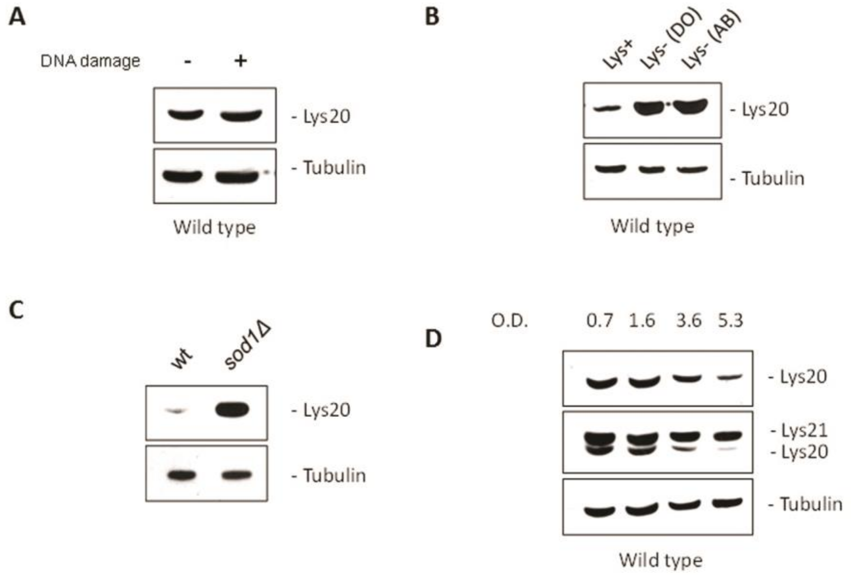

Figure S4. Lys20 protein levels were affected by environmental conditions.

(A) Lys20 levels were unaffected by DNA damage. Lysates of wild-type cells treated with 0.1M Hydroxyurea for 90 minutes were compared to untreated cells. **(B)** Lys20 expression in wild-type cells was affected by available lysine. The lys+ lane corresponds to cells grown in YPAD, the lys- (DO) lane indicates that the cells were grown in defined medium without lysine. Lys- (AB) means medium containing only the amino acids and nucleotides required by the auxotrophies of the strain tested: adenine, histidine, leucine, uracil and tryptophan. **(C)** Lys20 expression was increased in *sod1Δ* strains. *SOD1* null strains have increased levels of oxidative stress due to inability to detoxify superoxide (73). **(D)** Wild-type cells grown to high densities had reduced levels of Lys20. The measured A_{600} value of the lysates tested is indicated on the top. Lane 1 (0.7) indicates logarithmic phase growth. The blots labeled Lys20 were probed with the mAb 40C4, whereas the blots labeled Lys20, Lys21 were probed with mAb 36C3, which recognizes both proteins. All blots were re-probed for tubulin as a loading control.
